# The Effect of Rhythmic Tactile Stimuli Under the Voluntary Movement on Audio-Tactile Temporal Order Judgement

**DOI:** 10.3389/fpsyg.2020.600263

**Published:** 2021-02-09

**Authors:** Taeko Tanaka, Taiki Ogata, Yoshihiro Miyake

**Affiliations:** ^1^Department of Computational Intelligence and Systems Science, Tokyo Institute of Technology, Yokohama, Japan; ^2^Department of Computer Science, Tokyo Institute of Technology, Yokohama, Japan

**Keywords:** voluntary movement, rhythmic stimuli, tactile, temporal order judgment, just noticeable difference

## Abstract

The simultaneous perception of multimodal sensory information is important for effective reactions to the external environment. In relation to the effect on time perception, voluntary movement and rhythmic stimuli have already been identified in previous studies to be associated with improved accuracy of temporal order judgments (TOJs). Here, we examined whether the combination of voluntary movement and rhythmic stimuli improves the just noticeable difference (JND) in audio-tactile TOJ Tasks. Four different experimental conditions were studied, involving two types of movements (voluntary movement, involuntary movement) and two types of stimulus presentation (rhythmic, one-time only). In the voluntary movement condition (VM), after the auditory stimulus (cue sound) participants moved their right index finger voluntarily and naturally, while in the involuntary movement condition (IM), their right index finger was moved by the tactile device. The stimuli were provided in a rhythmic or one-time only manner by hitting inside the first joint of the participants' right index finger using a tactile device. Furthermore, in the rhythmical tactile (RT) conditions, tactile stimuli were presented rhythmically to the right index finger 5 times consecutively. On the other hand, in the one-time tactile (1T) conditions, tactile stimuli was presented one-time only to the right index finger. Participants made an order judgment for the fifth tactile stimuli and the first and only auditory stimuli. In our TOJ tasks, auditory-tactile stimulus pairs were presented to participants with varying stimulus-onset asynchronies (SOAs; intervals between the within-pair onsets of the auditory and tactile stimuli). For the two stimuli presented at a time that were shifted by the SOA, the participants were asked to judge which one was presented first, and they were given a two-choice answer. Using a non-parametric test, our results showed that voluntary movement and rhythmic tactile stimuli were both effective in improving the JNDs in TOJ Tasks. However, in the combination of voluntary movement and rhythmic tactile stimuli, we found that there was no significant difference in JNDs in our experiments.

## Introduction

To perceive the external environment our brain uses multimodal sensory information that includes data derived from visual, auditory, and tactile perception. The key to robust perception is the combination and integration of multiple sources of sensory information (Ernst and Bülthoff, 2004). Moreover, when we integrate the information subjectively, we can understand it as a single event precisely, despite delays in the arrival of the information. The simultaneous perception of multimodal information is important for efficient interactions with the environment or other people (Slutsky and Recanzone, 2001; Meredith, 2002; Jörg and Rainer, 2003). In reality, during the integration processing of sensory information, various delays occur in the feedback from the environment and auditory sensation, and we perceive the world using multimodal sensory information from the external environment and integrate the information subjectively.

Many previous studies have focused on the simultaneous perception of multimodal information. The first important factor identified in previous studies was voluntary movement. Haggard et al. (2002) found that the time lapse between active movement and the sensory feedback of the movement is perceived to be shorter than passive movement and its feedback. Recent studies suggest that voluntary movement affects the temporal order judgment (TOJ) task of sensory stimuli in multisensory integration. Shi et al. (2008) performed the TOJ task in visual-haptic integration under two conditions: passive-movement and voluntary movement. They demonstrated a reduction in just noticeable differences (JNDs) by active hand movements. In addition, other studies have shown that JNDs under voluntary movement conditions are statistically lower than those under involuntary movement and no movement conditions (Nishi et al., 2014; Kitagawa et al., 2016). These studies indicate that voluntary movement itself has some influence on simultaneous perception.

The second factor is the way in which the stimuli are presented. Rhythms have the property of making people predictable (Thaut, 2008; Thaut et al., 2015). Rhythmic stimuli contribute to a prediction, and it is thought that this improves the time perception between multimodal sensory information. In visual stimuli, previous studies have reported an increase in the temporal resolution of simultaneous perception when the visual information leading to the prediction of object collisions was given in succession (Correa et al., 2006). This is thought to be the result of the cyclic presentation of visual stimuli, which creates a rhythm that makes the stimuli predictable. In auditory stimuli, a previous study by Thaut et al. (1993) showed that in a synchronized tapping task with a single sensory modality, performed with periodic changes in the period of the sound stimulus, the response period followed the stimulus cycle one step later. This result suggests that subjects can predict the upcoming stimulus presentation time using the previous stimulus cycle as a guide. In addition, the temporal expectation or selective temporal attention improves tempolas aspects of performance from early perception to response such as temporal discrimination and reaction time (Nobre and Rohenkohl, 2014). On the other hand, the experiments of other previous multisensory studies regarding the TOJ task reported that sensory-based predictions did not improve the time of occurrence (Kitagawa et al., 2016). The authors tried to determine whether the sensory-based prediction was responsible for the improvement. However, their predictable condition was an auditory sequence with 500-ms intervals presented as an auditory anticipatory cue, which was longer than that reported in a previous study (Halpern and Darwin, 1982). Hence, they concluded that no improvement in temporal sensitivity was observed. These results suggest the possibility that the time prediction and rhythmic tactile stimuli can affect the JND during the TOJ task. However, the existence and extent of their effects are still to be explored. In the present study, we examined whether rhythmic tactile stimuli were effective in improving the value of JNDs.

Furthermore, we examined a combination of voluntary movement and rhythmic tactile stimulus presentation. These two factors have already been reported by previous studies to be related to improvements in the accuracy of JNDs in TOJ Tasks. However, it is not yet clear how to influence and improve the JNDs through the combination of voluntary movement and rhythmic stimulus presentation. Here, in order to explore the relationship and effectiveness of this combination, we performed the TOJ task between the auditory and tactile stimuli, with the rhythmic stimuli being presented by the latter. Moreover, we confirmed whether the JNDs were improved by the combination of the conditions with voluntary movements and rhythmical tactile stimuli. The JNDs were used as the validation index for our experiments, and comparative verifications were performed from the viewpoint of which condition improved the temporal resolution.

## Methods

### Participants

Seventeen healthy Japanese volunteers participated in this experiment (4 females and 13 males; average age: 27.0 years; range: 23–30 years). All participants were right-handed with normal auditory thresholds and senses of touch. They did not exhibit any difficulty moving their right index fingers. Moreover, none of them had previously participated in a TOJ task. Informed consent was obtained from all participants prior to their participation in the experiment. The participants were paid for their participation, and the experiment was approved by the ethics committee of the Tokyo Institute of Technology.

### Experiment Setup

**Figure 1:**
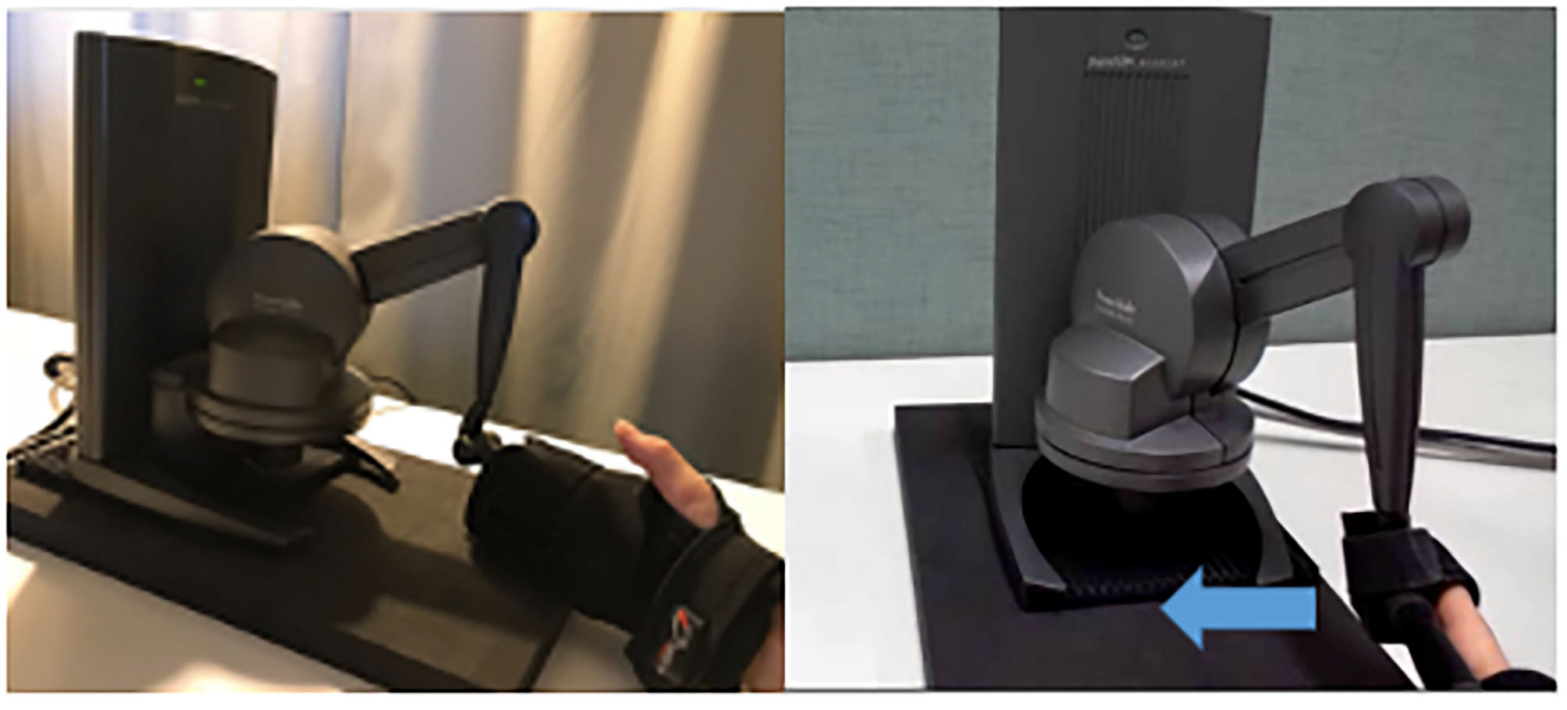
Experiment setup. The tactile stimulus was an impulse force consisting of a rectangular pulse (5 N, 10 ms) applied to the participant's the first joint of the right index finger on the palmar side. This tactile device was attached to the participant's right hand. The participants anchored with their elbows on the desk. After the cue sound, they started their motion voluntarily, naturally and horizontally in the right to left in the VM condition. In the IM condition, first, participants kept the position on the desk. After the cue sound, the right hand of participant was moved by the device.

The tactile stimulus was an impulse of force consisting of a rectangular pulse (5 N, for 10 ms) delivered to the participant's right index finger on the palmar side, orthogonal to the finger movement. We used a PHANToM® Desktop haptic device (SensAble Technologies, Woburn, MA, USA). It consists of a pen type control unit on a link mechanism arm. It is capable of 3 or 6 degrees of freedom of force presentation and position and angle measurement. Furthermore, the device operates with an update cycle of >1,000 Hz, which is desirable for haptic devices. This in turn facilitates the presentation and measurement of the reaction force at a time resolution of 1 KHz through the reaction force presentation arm and a measurement of the arm tip position with a spatial resolution of 0.023 mm (Figure 1). The timing of stimulus presentation and device movement was controlled with an error margin of 1 ms.

The sound delivered was a sinusoidal wave (2,000 Hz, 50 dB, for 10 ms). These sensory stimulation systems were operated by computer programs installed on a PC workstation (HP xw4600/CT; Hewlett-Packard, Palo Alto, CA, USA), and were developed using the Open Haptics software development toolkit (SensAble Technologies) on the Microsoft Visual C++ 2008 platform (Microsoft, Redmond, WA, USA).

### Task and Conditions

The following two tasks are often used while investigating simultaneous perception: simultaneity judgment (SJ) tasks (Schneider and Bavelier, 2003; van Eijk et al., 2008) or TOJ tasks (Spence et al., 2001; Zampini et al., 2003; van Eijk et al., 2009). In the SJ task, two stimuli are presented at various stimulus onset asynchronies (SOAs) and the participants answer whether the two stimuli are simultaneous or not. In a TOJ task, the participants judge the temporal order of the two stimuli. Previous studies reported the existence of the prior entry effect in the TOJ task (Spence et al., 2001; Zampini et al., 2005). Accordingly, in our experiment we considered to direct participants' attention to one of the stimuli in order to avoid being affected by prior entry effects. We instructed the participants to pay attention to auditory stimuli and asked them auditory stimulus had come first or not. Therefore, participants indicated which stimulus occurred first using a two-alternative forced-choice procedure. They used a foot pedal for providing their responses.

We conducted the experiment under a total of four conditions. Regarding the type of movement, there were two conditions: voluntary movement (VM) and involuntary movement (IM). Additionally, two methods of presenting the stimulus were set: rhythmic tactile stimuli (RT) presented multiple times (5 times) consecutively, or one single presentation (1T). Participants in the 1T condition judged the temporal order of the auditory and tactile stimulus presented once. In contrast, those in the RT condition judged the order for the rhythmically presented fifth tactile stimuli and the first auditory stimulus. We represent the combination of the type of movement and rhythm conditions using a hyphen. For example, the combination of the VM and RT conditions is described as a VM-RT condition. In addition, we had set nine types of time intervals (SOA of ±240, ±90, ±60, ±30, and 0 ms) as the deviation of the presentation time for the auditory and tactile stimulus. The positive SOA values indicated presentation of the auditory stimulus before the tactile stimulus, while negative values indicated that the tactile stimulus had been presented first. A value of 0 indicated physical synchrony. We requested the participants to indicate the individual temporal order of the two stimuli shifted by the SOA value under all conditions.

### Procedure

The experiment was conducted in a darkened, sound-attenuated room. The participants were asked to put on sound-insulating earmuffs to control ambient noise to the maximum extent possible. The auditory stimulus presented was white noise (50 [dB], 15 [ms]), which is thought to be rarely affected by echo, through earphones in both ears. It was considered to be rarely affected by echo. The participants were seated in front of the stimulation systems, with the palm of the right hand and the index finger on the desk. The PHANToM device was placed such that the tactile stimuli could hit the palmar side of the right index finger. All subjects were informed on how to use the device.

**Figure 2:**
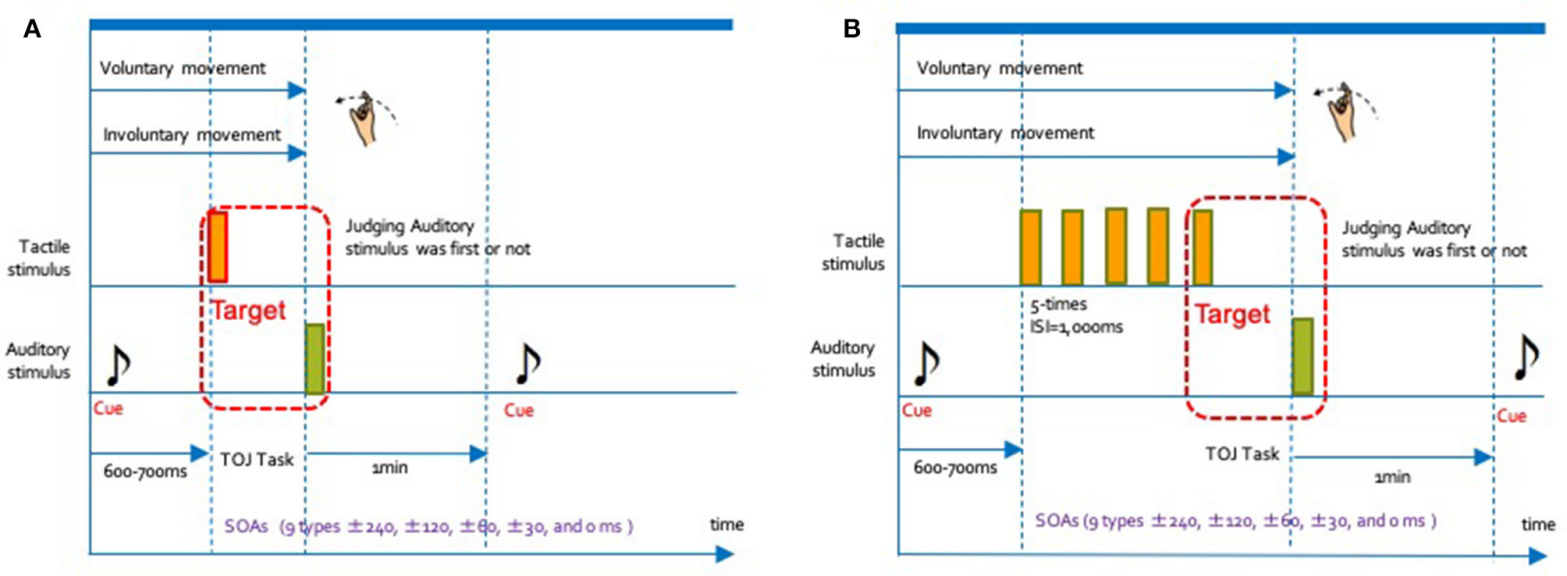
Schematic flow chart for one trial in each of the 4 conditions. We conducted the experiments in the manner shown in this chart. The interval between the cue and the TOJ task was randomly set from 600 to 700 ms. The interval between trials was 1-min. **(A)** 1 tactile stimulus (1T): voluntary movement condition (VM), in which participants voluntarily started to move their right index fingers; Involuntary movement condition (IM), in which the tactile device moved the participants' right hand and index fingers. **(B)** Rhythmical tactile stimuli (RT): Voluntary movement condition (VM), in which participants voluntarily moved their right hands and index fingers together 5 times; involuntary movement condition (IM), in which the tactile device moved the participants' right hands and index fingers together 5 times.

Figure 2 shows the schematic flow chart for one trial of our experiment. At the beginning of each condition, the one auditory stimulus (cue sound) indicating initiation of the enforcement, was presented in both ears through headphones. After the cue sound, participants started their motion in the VM condition. In the IM condition, they were moved by the tactile device. Then, two types of stimuli were presented: the first stimulus (either tactile or auditory) was delivered with a random delay of 600–700 ms after the cue sound onset. The second stimulus (distinct from the first) followed the first one after one of the nine SOAs described above. Each of the auditory and tactile stimuli lasted for 10 ms.

Participants were asked to keep their elbows in a fixed position on the desk and to move their index finger horizontally from the right to left. They attended practice sessions of a maximum of 30 repetitions in each condition before the commencement of the formal experimental trial. This facilitated their familiarization with the device and the TOJ task. For each condition, the practice ended when the participants said they were ready to start. We presented three measurement blocks in each condition after the practice session. They performed each of the nine SOAs trials once under each condition in one and three blocks consecutively. Thus, they completed 27 trials for each condition. The SOAs were presented in a random order within each block. Moreover, white noise was played in the background to effectively mask any sounds made by the haptic device. The participants used the foot pedal (as described above) to decide which of the two stimuli was presented first. We did not inform them about the stimulus that had occurred first in each condition.

#### Voluntary Movement Condition

For the VM condition, participants were attached to the force-sensing PHANToM device and they were instructed to move their index finger voluntarily and naturally after an audible cue. They moved the index finger horizontally from right to left and from the fully extended position to completely flexed to the palm side. This allowed the tactile stimuli to hit the underside of the right index finger. In the 1T condition, tactile stimuli were presented only once. Participants judged the temporal order of the stimuli for auditory and tactile stimuli presented one at a time. In the RT condition, participants move their index finger voluntarily five times, tactile stimuli were presented to the right index finger rhythmically each times in response to spontaneous movements. The order of the fifth rhythmically presented tactile stimulus and the first auditory stimulus was judged. We provided a short interval of 1 min between the blocks, following the complete cessation of the movement in each block.

#### Involuntary Movement Condition

In the IM condition, participants remained stationary when starting each trial. They stayed with his elbows on the desk, the palmar side of their right index fingers held on the tactile device. After an audible cue, their right-hand and index finger were moved by the device. Similar to the VM condition, the first stimulus (either tactile or auditory) was presented with a random delay (600–700 ms) after a sound cue. The procedure for evaluating the temporal order of the two stimuli were the same as in the VM condition.

### Data Analysis

We used the MATLAB Statistics Toolbox (MathWorks, Natick, MA, USA) for the statistical regression calculations and graphic representation of the results. First, we calculated the proportion of the answers given for each SOA in which the auditory stimulus was perceived first. From the response data that we collected, we excluded the data for practice time for analysis. Logistic regression using a generalized linear model was conducted for the ratio data.

The following equation was applied to the regression analysis, where *y* represents the percentage of correct answer and *x* denotes the value of the SOA:

(1) $$\begin{array}{l} y=\frac{1}{1+e^{\frac{\left(\alpha -x\right)}{\beta }}} \end{array}$$

By using β obtained from the regression analysis, we defined the JND by the following equations:

(2) $$\begin{array}{l} \text{JND}=\frac{{{\text{x}}_{75}-\text{x}}_{25}}{2}=\text{β}\log3 \end{array}$$

The values of JND were calculated for each participant in the regression analysis based on two equations (Finney, 1952).

Here, β is related to the JND, and *x*_*p*_ represents the SOA, with p being the percent of “auditory stimulus came first” responses. The JND was defined as one-half of the SOA where the percentage of participants reporting “auditory stimulus came first” is 75 and 25%, respectively. We determined the JND values for each participants using regression analysis and processed the data statistically to obtain the mean and standard error values for each condition.

The lower the JND, the narrower the discrimination threshold of the pair of stimuli, indicating that the distinction accuracy in terms of time is high. The data were analyzed, and we statistically tested for each of the four conditions (IM-1T, IM-RT, VM-1T, and VM-RT). Statistical analysis was performed using the Statistical Package for Social Scientists version 26 (SPSS; IBM Corp., Armonk, NY, USA) and R (version 4.0.3). Since the data to be analyzed did not follow a normal distribution, a non-parametric test was performed.

## Results

**Figure 3:**
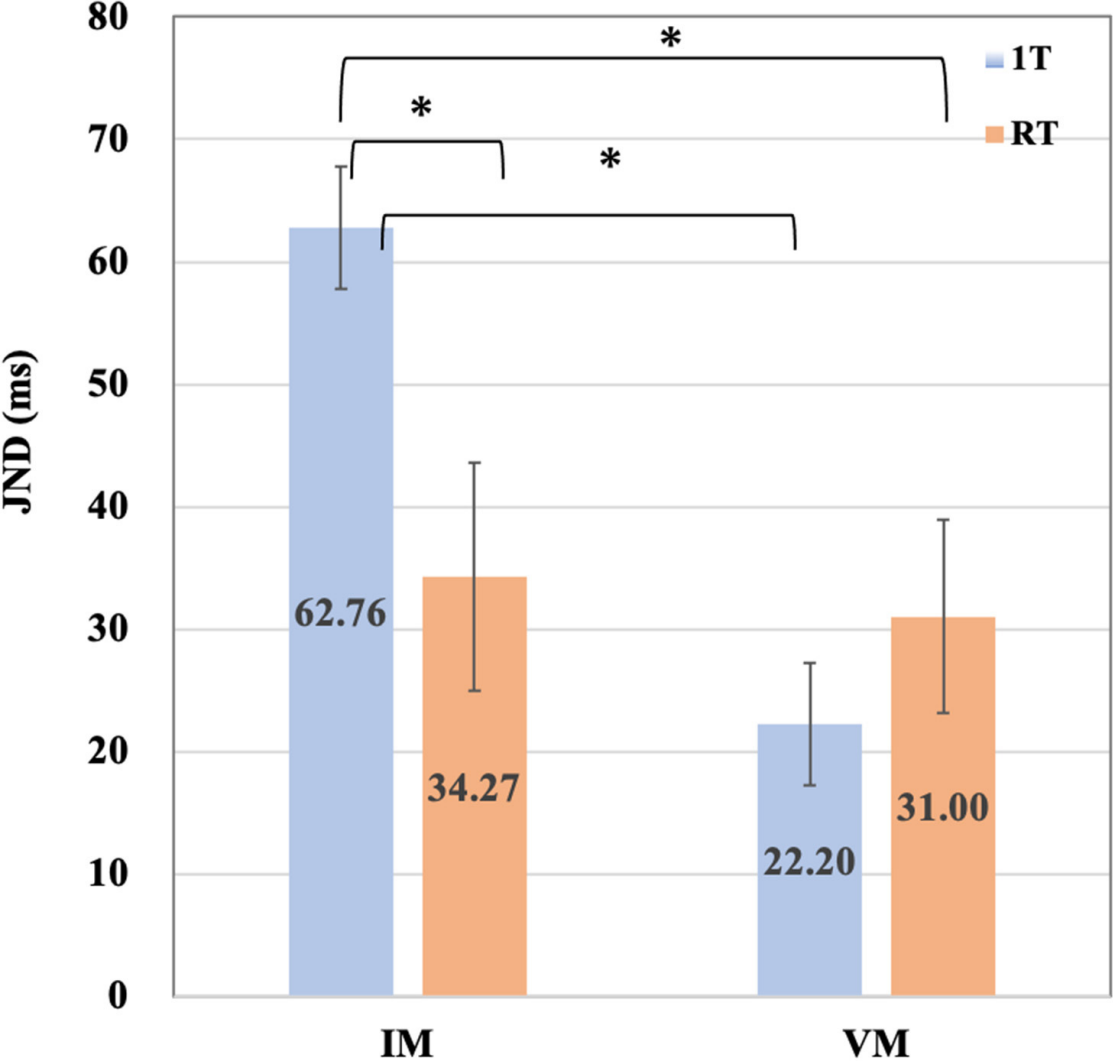
The means and error bars of JNDs for the four conditions. The means and error bars for the four conditions datasets by 17 participants are shown in the bar graph. It represent significant difference. *p < 0.05 (Wilcoxon signed rank exact test).

**Figure 4:**
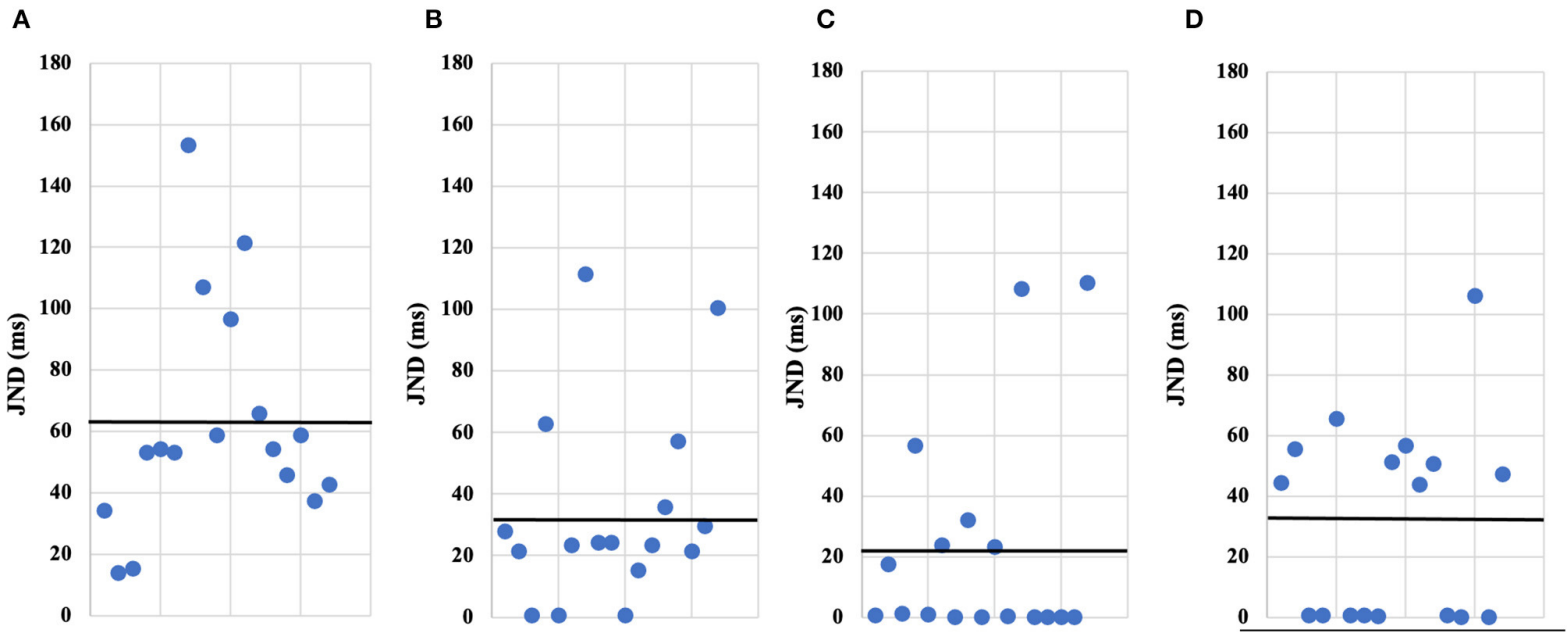
The JNDs by individual of participant's in each the conditions. The scatterplots **(A–D)** show the JNDs by individual of participant's in each the conditions of our experiments; **(A)** IM-1T condition, **(B)** IM-RT condition, **(C)** VM-1T condition, **(D)** VM-RT condition. Each point is an individual's JND value, also the black horizontal line is the average JNDs value.

Table 1 summarizes the means and standard errors of the JND values for each of the four conditions. Figure 3 contains a bar chart depicting the JND mean for each condition. In contrast, the distribution of the individual data of the participants recorded in each of the four conditions is shown in a scatterplot (Figure 4).

**Table 1:** The JND values under the four conditions.

**Condition name**		**IM-1T**	**IM-RT**	**VM-1T**	**VM-RT**
JND	Mean	62.76	34.27	22.20	31.00
	Std. Error	8.97	7.74	8.84	7.89

*IM, involuntary movement; VM, voluntary movement; RT, rhythmic tactile stimuli; 1T, one-time tactile stimulus; JND, just noticeable difference*.

The data did not follow a normal distribution. Therefore, we conducted the corresponding non-parametric analysis. First, our data were evaluated using the Friedman rank sum test comparing four conditions (IM-1T, IM-RT, VM-1T, and VM-RT), which yielded the following results: chi-squared = 8.3647, *df* = 3, *p* = 0.039. It confirmed a significant difference. Second, we conducted the Wilcoxon signed rank test for the paired comparisons of all conditions with the JNDs. The paired comparison of all conditions using Wilcoxon signed rank test with Benjamini-Hochberg (BH) correction adjustment revealed the significant difference (*p* < 0.05) between IM-RT and IM-1T (*p* = 0.038), IM-1T and VM-1T (*p* = 0.039), and IM-1T and VM-RT (*p* = 0.040). Table 2 summarizes the results of the paired comparison. We found no substantial difference between VM-RT and VM-1T (*p* = 0.516).

**Table 2:** Paired comparisons of all conditions using Wilcoxon signed rank test.

**Pair condition name**		**Sig**.	**Adj. Sig**.	**W**	**Cliff's delta**
IM-1T	VM-1T	0.0093	0.0386	130	0.668 (large)
IM-RT	VM-RT	0.8536	0.8536	72	0.093 (negligible)
IM-1T	IM-RT	0.0129	0.0386	25	0.505 (large)
VM-1T	VM-RT	0.4307	0.5168	94	0.273 (small)
IM-1T	VM-RT	0.0202	0.0403	28	0.488 (large)
VM-1T	IM-RT	0.1454	0.2181	108	0.419 (medium)

*Friedman test and pair comparison of four conditions using Wilcoxon signed rank test with BH correction adjustment showed the significant difference (P < 0.05) between IM-1T and VM-1T (p = 0.039), IM-RT and IM-1T (p = 0.038), IM-1T and VM-RT (p = 0.040). Effect size estimates were calculated using Cliff's delta*.

## Discussion

Several previous studies have focused on the simultaneous perception of multimodal information under voluntary movements, or under the modality of rhythmic sensory input. However, two points remained unclear: how can we improve the JNDs by presenting rhythmic tactile stimuli, and how effective is the combination of voluntary movements and rhythmically presented stimuli for improving the JNDs?

In the present study, we examined the effect of rhythmic tactile stimuli, and the combination of voluntary movement and rhythmic tactile stimuli in improving the value of JND. We then performed TOJ tasks involving audio-tactile integration under four conditions (IM-1T, IM-RT, VM-1T, and VM-RT). Below, we discuss the results of the experiments that examined the combination of voluntary movement and rhythmic stimulus presentation.

We first evaluated the effect of rhythmic tactile stimuli. In our experiments, the values of JNDs in the IM-RT condition were significantly lower than those in the IM-1T condition. This suggests that the rhythmic tactile stimuli under the IM condition are effective in improving the JNDs. In previous studies, the rhythmic and repeated visual stimuli improved time perception (Correa et al., 2006; Vroomen and Stekelenburg, 2010). Furthermore, we demonstrated that rhythmic and repeatedly presented tactile stimuli can improve the value of JNDs, and replicated the effect seen by the rhythmical visual stimuli. Taken together, our results showed that JND was improved by the tactile rhythmic stimuli. The improvement of JNDs by rhythmical tactile stimuli could be caused by temporal prediction or selective temporal attention. The temporal regularity and predictability of events manipulated through rhythms, hazard functions, and cues enhanced perceptual sensitivity and modulated the primary visual and auditory cortex (Nobre and Rohenkohl, 2014). Thus, the modulation of the somatosensory cortex by rhythmic tactile stimuli could have improved the temporal resolution in our audio-tactile TOJ task. The neural mechanism by which rhythmic stimulation improves JND should be investigated in the future.

The decrease of the JNDs by rhythmic tactile stimuli would be affected by the interval of the rhythmic stimuli. Baumgarten et al. (2017) presented an subliminal electrotactile stimulus 20–600 ms before two subliminal electrotactile stimuli presented in short interval, for a discrimination perception task. The discrimination accuracy was modulated rhythmically in the beta-band (13–18 Hz). This result suggests that the temporal perception was related to the phase of neuronal oscillation in the beta-band in the parieto-occipital or primary somatosensory cortex. Thus, the effect of rhythmic tactile stimuli on the JNDs in the TOJ task could also be modulated rhythmically along with the intervals of the rhythmic stimuli. This is another possibility for future work.

Next, we analyzed the effectiveness of the combination of VM and RT for improving JNDs. In our experiments, the VM-1T condition was the most effective in improving JNDs. On the other hand, in pairwise comparisons of all conditions, the analysis showed there was no significant difference between VM-RT and VM-1T. According to these results, although there were some individual differences, the VM-RT combination condition was likely affected by the ceiling effect, i.e., no further improvement in the accuracy of the JNDs in TOJ Tasks was possible.

Regarding the tactile stimuli, Vitello et al. (2006) reported that the motor state of the forearm became slower in the order of stationary, passive, and active states, respectively, when they measured the motion direction discrimination performance for tactile stimuli moving laterally on the index finger. They also showed that there was a decrease in tactile sensibility for direction discrimination during active arm movement. Furthermore, they suggested a general decrease in tactile sensibility by descending motor commands that cause an inhibition of ascending tactile information.

In conclusion, our results show that VM (voluntary movement) and RT (rhythmic tactile stimuli) were both effective in improving the JNDs in the TOJ tasks. However, in the combination of VM and RT, we found that there was no significant difference between VM-RT and VM-1T on JNDs in our experiments. Our experiments have revealed this for the first time using TOJ tasks. It is important to discuss the potential mechanisms underlying these results. However, they remain unclear. It is uncertain whether the ceiling effect under the voluntary movement condition affects only the rhythmical tactile stimuli or not. In future work evaluating the effect of the combination of each factor, namely the type of movement (VM vs. IM) and the type of stimulus presentation (RT vs. 1T), on the JNDs in TOJ Tasks, rhythmic stimuli should be presented in non-tactile modalities and be verified under voluntary movement conditions. Furthermore, regarding the improvement of JNDs by rhythmical tactile stimuli, it would be worthwhile to explore previous findings regarding the neural mechanisms underlying temporal expectations to incorporate more findings in the neural mechanisms.

## Data Availability Statement

The datasets for this article are not publicly available because Ethics Committee does not allow the disclosure of participants data. Requests to access the datasets should be directed to Taeko Tanaka, tanaka.t.bq@m.titech.ac.jp.

## Ethics Statement

This experiment was conducted with the approval of the Tokyo Institute of Technology's Research Ethics Review Committee. Informed consent was obtained from all participants.

## Author Contributions

TT conceived of the study and designed the experiments, collected data, carried out data analysis, and drafted the manuscript. YM designed and supervised the study and experimental design and provided conceptual advice. YM and TO provided advice regarding the analytical methods, results, and the manuscript. TO provided advice regarding analytical program. All authors revised the manuscript and gave final approval for publication.

## Conflict of Interest

The authors declare that the research was conducted in the absence of any commercial or financial relationships that could be construed as a potential conflict of interest.

## Abbreviations

VM: voluntary movement
IM: involuntary movement
RT: rhythmic tactile stimuli
1T: one-time tactile stimuli
TOJ: temporal order judgment
SOAs: stimulus-onset asynchronies
JND: just noticeable difference.
